# Mono-institutional retrospective cohort analysis of the insurance status dependent access to ENT-professionals and survival in head and neck squamous cell carcinoma

**DOI:** 10.1186/s12913-020-06035-2

**Published:** 2021-01-08

**Authors:** Andreas Knopf, Simon Teutsch, Henning Bier

**Affiliations:** 1 Otorhinolaryngology/Head and Neck Surgery, Faculty of Medicine, University of Freiburg, Killianstr. 5, 79106 Freiburg, Germany; 2 Otorhinolaryngology/Head and Neck Surgery, Ismaninger Str. 22, 81675 München, Germany

**Keywords:** Insurance status, outcome, survival, Head and neck squamous cell carcinoma, Socioeconomic status

## Abstract

**Background:**

To access the influence of insurance status on time of diagnosis, quality of treatment and survival in head and neck squamous cell carcinoma (HNSCC).

**Methods:**

This mono-institutional retrospective cohort analysis included all HNSCC patients (*n* = 1,054) treated between 2001 and 2011, and subdivided the cohort according to the insurance status. Differences between the groups were analyzed using the Chi square and the unpaired student’s t-test. Survival rates were calculated by Kaplan-Meier and Cox regression for forward selection.

**Results:**

Nine hundred twenty-five patients showed general, 129 private insurance. The 2 groups were equal regarding age, gender, tumor localization, therapy, and N/M/G/R-status. The T-status differed significantly between the groups showing more advanced tumors in patients with general insurance (*p *= 0.002). While recurrence-free survival was comparable in both groups, overall survival was significantly better in private patients (*p* = 0.009). The time frame between first symptom and diagnosis was equal in both groups.

**Conclusions:**

The time frame between subjective percipience of first symptom and final therapy did not differ between the groups. In our cohort, access to otorhinolaryngological specialists is favorable in both, patients with general and private insurance. Recurrence-free survival was comparable in both groups, indicating successful HNSCC treatment both groups. However, overall survival was significantly better in patients with private insurance suggesting other socioeconomic factors influencing survival.

## Background

In the past decades, many therapeutic efforts were made to improve the prognosis of head and neck squamous cell carcinoma (HNSCC) patients. However, according to surveys of the Robert-Koch-Institute, the overall five-year survival in Germany is less than 50% [1]. Variables like age, gender, tumour location, and histological grading are known independent prognostic parameters. One main factor influencing patients’ survival is the insurance status. Many studies analyse mortality and disease-modifying parameters related to specific country-related healthcare systems. Particularly, in countries without compulsory insurance, uninsured patients show advanced tumour stages at the time of diagnosis, hence poor survival [2–5]. Other countries such as Japan did not observe differences in survival parameters with the insurance status [6]. In Germany, there is a long history of compulsory insurance attributed to Bismarck’s governmental decision in 1883. However, different access to the healthcare system concerning general or private insurance status, and therefore prolonged diagnosis and treatment in patients with general insurance, is still a debate. While tumour stage at diagnosis is an important prognostic factor for HNSCC patients [7, 8], the correlation between the time from diagnosis to treatment (TTI) and overall survival has been statistically proven, indicating that early treatment leads to better survival. More recently, TTI longer than 40–50 days showed a significantly worse outcome [9, 10]. However, data exploring the interval between the first symptom and cancer diagnosis (TTD) are still lacking. The perception of the first symptoms is affected by many subjective factors. In a prospective study, Dekate et al. tried to identify parameters that lead to late diagnosis in HNSCC [11]. The location of the tumour had a high impact, with hypopharyngeal cancer being one of the last to show clinical symptoms. Other factors delaying the time to diagnosis were advanced age and male gender, while easy access and early presentation to a specialist significantly reduced time to diagnosis. Early cancer diagnosis, therefore, depends on one hand, on the time till patients consult specialists after noticing the first symptom (patients delay), and on the other hand, on the prompt action of the physician (professional delay). Shafer et al. showed an avoidable delayed diagnosis in 15% of HNSCC patients which was caused likewise by the patient and the doctor [12–14]. However, reasons for patients’ delay and especially the influence of socioeconomic factors on delayed diagnosis represent an underestimated and still insufficiently researched risk factor in the survival of HNSCC.

The current study analyses the insurance status as a risk factor for delayed access to ENT-professionals, delaying cancer diagnosis and therefore, reduced recurrence-free and overall survival.

## Methods

Between January 2001 and December 2011, all patients who were treated for HNSCC were included in a mono-institutional retrospective cohort study. Data collection was done by a hospital-based data-acquisition system (SAP®) and validated for all patients by the regional tumour registry. Follow-up of survival parameters was done for at least 5 years in all patients, last updated in 2018. Histological samples were analysed by at least 2 experienced pathologists. Carcinoma in situ and other histological subtypes were excluded. Disease-related data such as age at diagnosis, sex, tumour location, TNM-status (UICC 7th edition), treatment modalities, and resection status were retrospectively collected. The period from the first symptoms until diagnosis was estimated in weeks. Patients were divided into 2 groups: patients with general (1) and private (2) insurance. Overall and recurrence-free survivals were analysed for all patients. Patients with lacking data, incomplete staging, and refused/incomplete treatment were excluded from survival analysis. Differences between the two groups were analysed using the Chi-squared test. Fisher’s exact test and the unpaired student’s t-test were used for categorical and continuous variables, respectively. Survival rates and curves were calculated and illustrated by the Kaplan-Meier method and further analysed by the log-rank test. Parameters with potential impact on survival were analysed with Cox regression for further selection. *P*-values < 0.05 were considered statistically significant. Statistical analysis was done using SPSS (SPSS Inc., Chicago, IL).

### Ethical considerations

Clinical data were collected retrospectively from the daily ENT routine. Patient data were analysed pseudonymised and published anonymised. The study was approved by the local Ethics Commission, Technical University Munich (No. 104/18S).

## Results

### Epidemiology of the HNSCC cohort

A total of 1,054 patients with HNSCC were included in our study with 925 and 123 patients having general and private insurances, respectively. Subgroup analysis of insurance status did not show differences in age and gender distribution (*p* = 0.06; *p* = 0.19; Table 1). The mean age at diagnosis was 60 and 62 years with a striking predominance for male patients (79%, 84%; Table 1). In both groups, oropharyngeal HNSCC (39%; 40%) represented the most frequent tumour site, followed by hypopharyngeal (21%, 21%), laryngeal (20%; 21%), and oral (15%; 11%) cancers (Table 1). Nasopharyngeal and sinonasal cancers, as well as cancer of unknown primary (CUP), accounted for < 5% in each subgroup. There were significant differences in the T status. While 418 patients (45%) with general insurance showed T3/4 status, 41 patients (32%) with private insurance had an advanced T status (32%) (*p* = 0.002; Table 1). Lymph node positivity was demonstrated in 60% and 61% of the underlying subgroup (*p* = 0.86; Table). The vast majority of patients (96–98%) had no distant metastasis at the time of diagnosis (*p* = 0.76; Table 1). A high percentage of regional lymph node metastasis in both groups with a UICC stage 4 disease in 513 (56%) and 63 (49%) patients with general and private insurances, respectively (*p* = 0.14; Table 1). In both groups, 18% of patients underwent primary surgery without adjuvant treatment. Surgery with adjuvant chemo-/radiation was applied in 433 (47%) and 69 (54%) patients with general and private insurances, respectively. Primary chemo-/radiation was recommended in 328 (35%) patients with general insurance and 37 patients (29%) with private insurance (*p* = 0.31; Table 1).

**Table 1:** Epidemiologic data of a typical HNSCC cohort, subdivided due to the insurance status. CUP: Cancer of unknown primary; C-/RT: Chemo-radiation

	General insurance	Private insurance	*p*-value
**n**	925	129	
**Age (years)**			0.06
Median	60	62	
Mean ± SD	60 ± 10	62 ± 11	
**Sex, n (%)**			0.19
Male	731 (79)	108 (84)	
Female	194 (21)	21 (16)	
**Location, n (%)**			0.52
Sinonasal	32 (4)	3 (2)	
Nasopharynx	14 (2)	5 (4)	
Oropharynx	363 (39)	52 (40)	
Hypopharynx	192 (21)	27 (21)	
Larynx	180 (20)	27 (21)	
Oral cavity	140 (15)	14 (11)	
CUP	4 (< 1)	1 (1)	
**T stage, n (%)**			0.002
T1/2	503 (54)	87 (67)	
T3/4	418 (45)	41 (32)	
**N stage, n (%)**			0.86
N0	366 (40)	50 (39)	
N+	559 (60)	79 (61)	
**M stage, n (%)**			0.76
M0	886 (96)	127 (98)	
M1	39 (4)	2 (2)	
**Grading, n (%)**			0.20
G1	39 (4)	5 (4)	
G2	452 (49)	53 (41)	
G3	407 (44)	62 (48)	
G4	27 (3)	5 (7)	
**R status, n (%)**			0.24
R0	510 (83)	78 (80)	
R1	52 (9)	6 (6)	
R2	11 (2)	3 (3)	
Rx	40 (7)	10 (10)	
**UICC stage, n (%)**			0.14
UICC I	136 (15)	21 (16)	
UICC II	117 (13)	17 (13)	
UICC III	152 (16)	26 (20)	
UICC IV	513 (56)	63 (49)	
**Therapy, n (%)**			0.31
Surgery	164 (18)	23 (18)	
Surgery + C/RT	433 (47)	69 (54)	
pC/RT	328 (35)	37(29)	

### Insurance status and survival in HNSCC

**Fig. 1:**
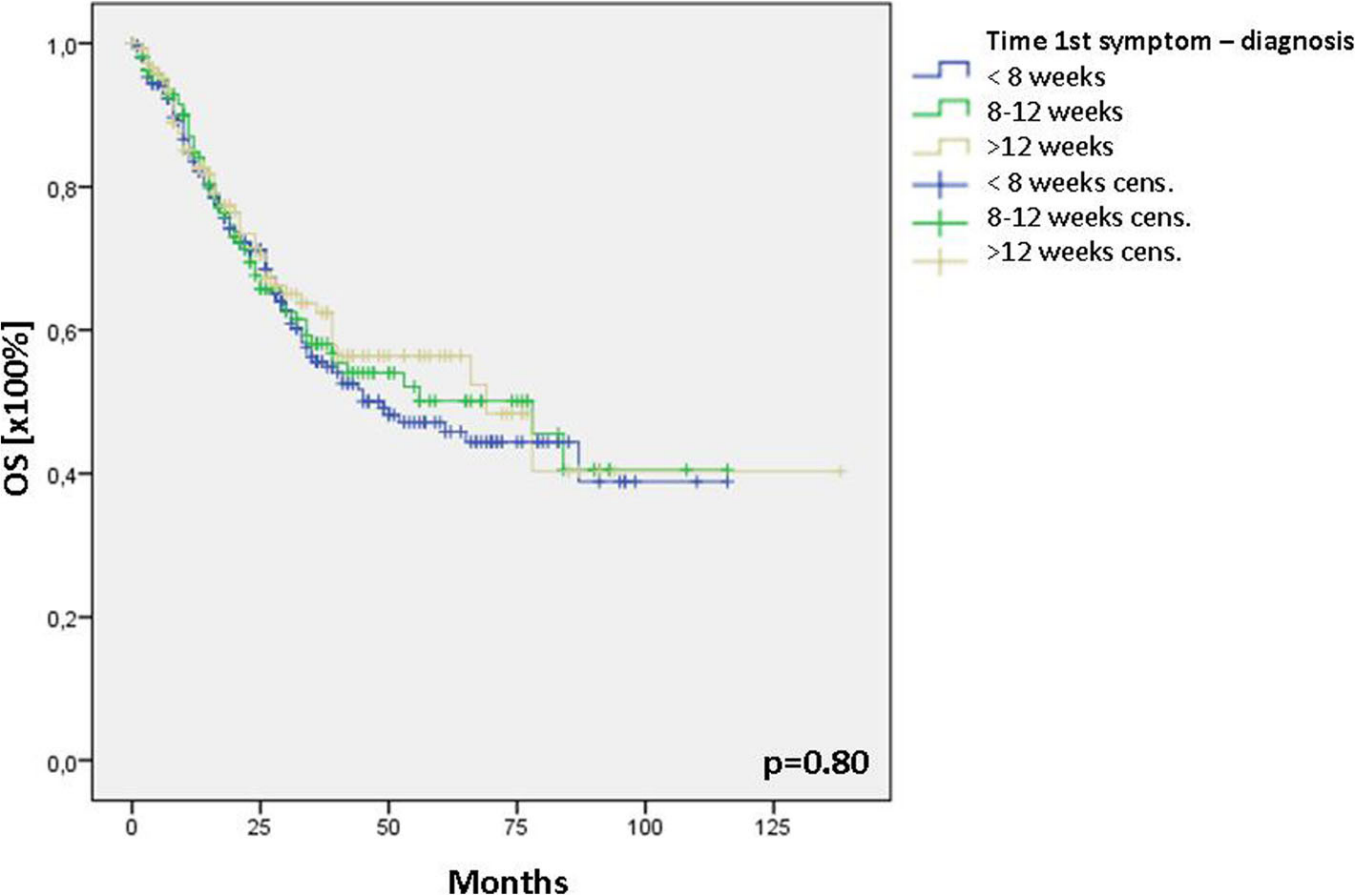
Overall survival after stratification of the time frame first symptom – final therapy

**Fig. 2:**
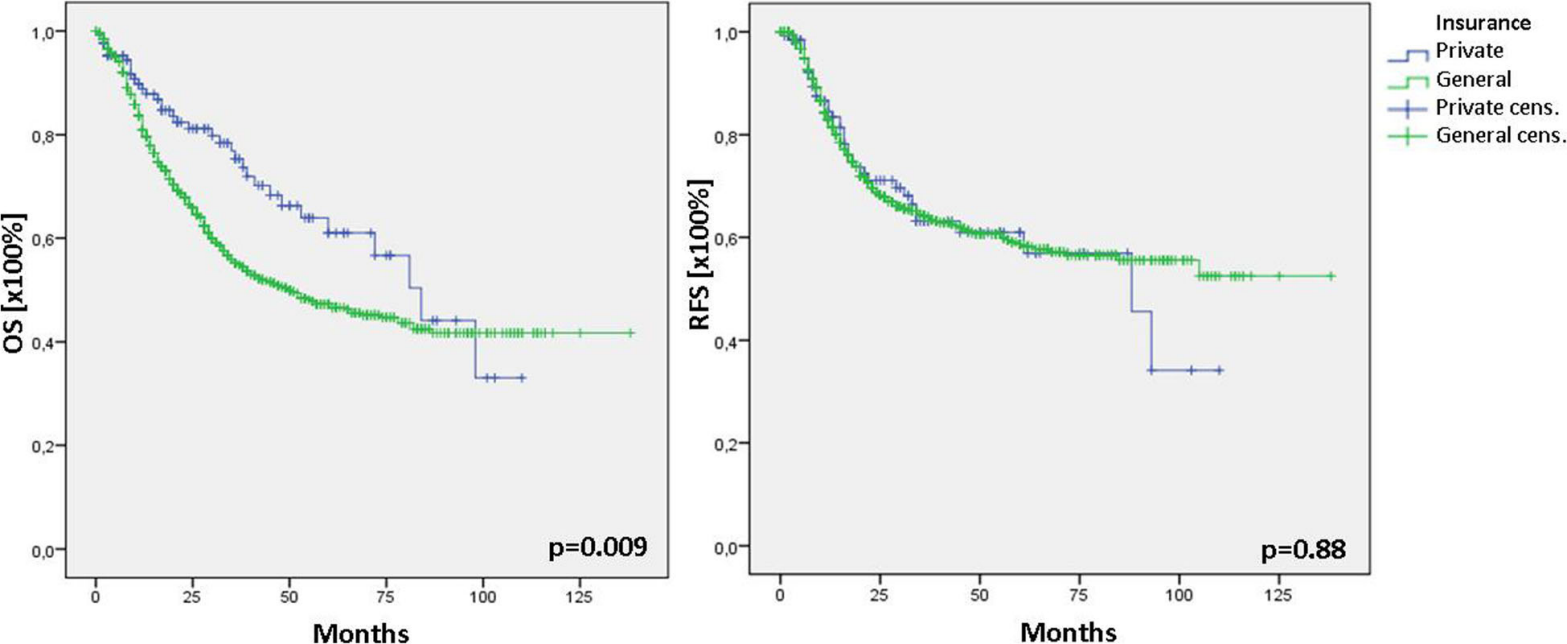
Overall and recurrence-free survival after stratification of the insurance status

The time between the first symptom and final therapy was hypothesised to influence survival in HNSCC. The entire cohort was stratified into 3 groups according to the time frame of the first symptom to final therapy (< 8 weeks, 8–12 weeks and > 12 weeks). In patients with a time frame < 8 weeks, the mean time between the first symptom and final therapy was 4.1 ± 1.6 weeks (median: 4 weeks), 9.9 ± 1.8 weeks (median: 10 weeks) in the group of 8–12 weeks, and 27.0 ± 12.3 weeks (median: 25 weeks) in patients with time frame > 12 weeks. Survival analysis did not reveal differences between the analysed groups (Fig. 1). Analysis of mean and median time from first symptom to final therapy showed no differences between patients with general and private insurance (*p* = 0.23). The mean time in patients with general insurance was 12.0 ± 11 weeks (median: 8 weeks) and 13.0 ± 13 weeks (median: 8 weeks) in those with private insurance. Subgroup analysis of overall survival due to different insurance status showed significant differences between the groups. While patients with general insurance showed a median overall survival of 50 months [95% CI: 36–64], patients with private insurance showed a significantly better median overall survival of 84 months [95% CI: 66–102] (*p* = 0.009; Fig. 2). Cox regression for further selection, analysing symptom duration, T-, and N-status, identified both, advanced T (T1/2 vs. T3/4; *p* < 0.0001; HR: 2.2) and N (N0 vs. N+; *p* < 0.0001; HR: 1.9) status being disease-modifying parameters (Table 2). Interestingly, there was no difference in the recurrence-free survival between the groups (*p* = 0.88; Fig. 2).

**Table 2:** Forward-selected Cox-regression identified increased T-status (T3/4) and lymph node positivity (N+) being associated with a 2.2 and 1.9-fold risk of disease associated deaths, while symptom duration did not impact survival. CI: confidence interval; HR: hazard ratio

	***p***-value	HR	95%-CI
Symptom duration			
<8 weeks vs. 8-12 weeks	0.37	0.9	0.6-1.1
8-12 weeks vs. >12 weeks	0.59	1.1	0.8-1.6
T1/2 vs. T3/4	<0.0001	2.2	1.6-3.1
N0 vs. N+	<0.0001	1.9	1.3-2.2

## Discussion

While many studies aim to stratify the overall HNSCC cohort by clinical and molecular markers, and therefore, try to predict treatment response, factors associated with the socioeconomic status were poorly explored [15, 16]. Low socioeconomic status is traditionally attributed to reduced overall survival [17]. Interestingly, recent studies showed higher mortality rates in breast, prostate, and thyroid cancers as well as melanoma in a high-income cohort, hypothesising that too much medical care results in over-diagnosis and unnecessary treatment [18]. However, information on the impact of socioeconomic status in HNSCC is poor. There are broad inter-relations between the socioeconomic and the insurance status. Many studies investigated the insurance status as an independent risk factor in overall survival. Regarding different healthcare systems worldwide, results differ significantly. Particularly in countries without compulsory insurance, uninsured patients show significantly increased tumour stages at diagnosis and poor survival [2–6]. In Germany, there is a long tradition of compulsory insurance. However, there is an ongoing debate on whether access to the healthcare system is aggravated for patients with general insurance. This might be of major impact because the prolongation of treatment after tumour diagnosis was proven to reduce patients’ survival [9, 19]. While the prolongation of treatment caused by different insurance status can hardly be analysed, we focused on primary access (first perception of symptoms to diagnosis) to ENT-professionals, an aspect that is often discussed as being prolonged in patients with general insurance. A total of 1,054 consecutively treated patients were included. Fourteen percent of the overall cohort had private insurance. The time interval between subjective symptom percipience and diagnosis was comparable in patients with general and private insurance, suggesting that there was no avoidable retardation of diagnosis generated by the health care system. Age and gender distribution as well as primary tumour location did not differ between the groups. However, patients with general insurance showed significantly advanced T status at the time of diagnosis. Underlying conditions resulting in different self-perception, as well as the attribution of early symptoms to severe illness, might be delusive at this point. In literature, a higher rate of patient’s delay was demonstrated in heavy smokers and drinkers [10]. Also, education, income, and social support seem to play an important role [20]. Accordingly, patients with private insurance demonstrated significantly improved overall survival when compared with their counterparts with general insurance. Interestingly, we could not observe differences in the recurrence-free-survival, suggesting successful cancer treatment in both groups. There might be different explanations for these contradictory results including therapy associated deaths due to late toxicity and cancer independent comorbidity in patients with general insurance. An association between health behaviour (especially smoking/drinking), socioeconomic status that influence oncologic outcome irrespective of cancer stage, and therapeutic strategies have recently been demonstrated [21]. Also, after including risk factors like smoking, alcohol abuse, and nutritional status as co-factors, studies still showed a high relevance of the socioeconomic status as an independent risk factor. In a prospective longitudinal study, Choi et al. identified low income, as well as low education, as predictors for poor survival with an impressive aberration of survival rates [22, 23]. Concomitant depression represents another often underestimated, independent risk factor in the outcome of HNSCC that is strongly associated with socioeconomic factors. The prevalence of preoperative depressive symptoms in HNSCC patients is high. Rieke demonstrated that 19% of patients with HNSCC suffer from pre- or post-therapeutic depression resulting in a 35% higher mortality [24]. Kim et al. showed a similar correlation between depression and mortality in patients with HNSCC; the incidence of depression was strongly associated with the pretreatment quality of life [25]. Also, the high influence of depression on post-treatment functional status and rehabilitation, as well as treatment adherence, has been shown in prospective studies [17, 26]. Even if prospective data regarding the relevance of depression as an independent risk factor still has to be completed, the demonstrated literature shows the relevance of the allowance and treatment of such comorbidities. The treatment of depression not only improves the quality of life but dramatically contributes to therapy success. Hence, it should be part of every follow-up care in the treatment of HNSCC.

## Conclusions

The time frame between the subjective perception of first symptoms and final therapy did not differ between patients with general and private insurances. However, patients with general insurance showed significantly advanced T status at the time of diagnosis when compared to those with private insurance. In Germany, access to otorhinolaryngological specialists is favourable in both patients with general and private insurance. Despite advanced T status in patients with general insurance, the recurrence-free survival was comparable in both groups, indicating successful HNSCC treatment. However, overall survival was significantly better in patients with private insurance suggesting other socioeconomic factors influencing survival.

## Data Availability

Data analysed from the daily ENT routine. The datasets generated and/or analysed during the current study are not publicly available because ethical approval does not include sharing raw data.
